# Supplementary material for: Characterizing the Mutational Landscape of Diffuse Large B-Cell Lymphoma in a Prospective Cohort of Mexican Patients
Source: Int J Mol Sci. 2024 Aug 28;25(17):9328. doi: 10.3390/ijms25179328 (PMC11394969; doi:10.3390/ijms25179328)
Supplement: Supplementary file 1 [file ijms-25-09328-s001.zip › ijms-3114367-supplementary.pdf]

**Supplementary Table S1. Genes comprising the custom panel**			
ARID1A	*DDX3X*	*KLHL14*	*REL*
ARID5B	*DTX1*	*KLHL21*	*RERE*
BCL10	*EP300*	*KLHL6*	*S1PR2*
BCL11A	*ETS1*	*KMT2D*	*SETD1B*
BCL2	*ETV6*	*MEF2B*	*SOCS1*
BCL6	*EZH2*	*MEF2C*	*SPEN*
BCL7A	*FOXC1*	*MIR17HG*	*SPIB*
BCOR	*GNA13*	*MPEG1*	*STAT6*
BTG1	*GRHPR*	*MTOR*	*TBL1XR1*
BTG2	*HASPIN*	*MYD88L265P*	*TMEM30A*
CCND3	*HLA-A*	*NFKBIA*	*TNFAIP3*
CD58	*HLA-B*	*NOL9*	*TNFRSF14*
CD70	*HLA-C*	*NOTCH1*	*TOX*
CD79B	*HLA-DMB*	*NOTCH2*	*TP53*
CDKN2A	*HNF1B*	*OSBPL10*	*TRIP12*
CHST2	*ID3*	*PIM1*	*TRRAP*
CIITA	*IL16*	*PIM2*	*UBE2A*
CREBBP	*IRF2BP2*	*PPP1R9B*	*VMP1*
CXCR5	*IRF4*	*PRDM1*	*VPS13B*
DDX3X	*IRF8*	*PRKCB*	*WEE1*

Supplementary Table S2. Driver mutations of patients with Diffuse Large B Cell Lymphoma				
**Patient ID**	**Gene**	**Mutation**	**Mutation Type**	**Protein Change**
DLBCL_001	EZH2	chr7:148508728 A>T	missense variant	Y646N
DLBCL_003	MYD88	chr3:38182641 T>C	missense variant	L260P
DLBCL_004	CREBBP	chr16:3778440-3778440 TGC>-	inframe deletion	QQ2202-2203Q
DLBCL_004	MTOR	chr1:11303294 C>T	missense variant	R430H
DLBCL_004	NOTCH2	chr1:120459205 C>T	missense variant	R2047Q
DLBCL_004	MEF2C	chr5:88024416 A>G	missense variant	S342P
DLBCL_004	NOTCH1	chr9:139418406 G>A	stop gained	R56*
DLBCL_011	TP53	chr17:7577539 G>A	missense variant	R248W
DLBCL_014	TP53	chr17:7577120 C>T	missense variant	R273H
DLBCL_014	NOTCH2	chr1:120459205 C>T	missense variant	R2047Q
DLBCL_014	EP300	chr22:41536164 C>T	missense variant	T594M
DLBCL_014	EP300	chr22:41566555 C>T	missense variant	R1478C
DLBCL_015	EZH2	chr7:148508728 A>G	missense variant	Y646H
DLBCL_017	TBL1XR1	chr3:176750884 G>A	stop gained	R431*
DLBCL_017	EZH2	chr7:148508728 A>T	missense variant	Y646N
DLBCL_018	TP53	chr17:7577539 G>A	missense variant	R248W
DLBCL_019	KMT2D	chr12:49433388 G>A	stop gained	R2687*
DLBCL_020	KMT2D	chr12:49434931 G>A	stop gained	Q2208*
DLBCL_020	TP53	chr17:7578508 C>T	missense variant	C141Y
DLBCL_020	NOTCH2	chr1:120468135 C>T	missense variant	R1435Q
DLBCL_020	BCL6	chr3:187446313 G>A	missense variant	R459C
DLBCL_020	EZH2	chr7:148515090-148515090 G>-	frameshift variant	P373X
DLBCL_020	NOTCH1	chr9:139401168 C>T	missense variant	G1301R
DLBCL_020	NOTCH1	chr9:139410064 G>A	missense variant	R592C
DLBCL_020	CDKN2A	chr9:21971053 G>A	missense variant	A102V
DLBCL_022	KMT2D	chr12:49424741 G>A	stop gained	R4536*
DLBCL_022	CIITA	chr16:11004092 G>A	missense variant	R956Q
DLBCL_022	CREBBP	chr16:3789685 G>A	stop gained	R1392*
DLBCL_022	CREBBP	chr16:3820660 G>A	stop gained	Q931*
DLBCL_022	NOTCH2	chr1:120458578 C>T	missense variant	R2256H
DLBCL_022	ARID1A	chr1:27106621 G>A	missense variant	E2078K
DLBCL_022	NOTCH1	chr9:139391833 G>A	missense variant	R2120C
DLBCL_022	NOTCH1	chr9:139396761 G>A	missense variant	R1783W
DLBCL_022	NOTCH1	chr9:139402690 G>A	stop gained	R1107*
DLBCL_022	BCOR	chrX:39934079 C>T	missense variant	D174N
DLBCL_023	ETV6	chr12:12022535 C>T	missense variant	P214L
DLBCL_023	MYD88	chr3:38182641 T>C	missense variant	L260P
DLBCL_026	CREBBP	chr16:3781324-3781324 AGG>-	inframe deletion	SL1680-1681L
DLBCL_026	MYD88	chr3:38182641 T>C	missense variant	L260P
DLBCL_028	KMT2D	chr12:49420463 G>A	stop gained	Q5096*
DLBCL_028	EZH2	chr7:148508727 T>G	missense variant	Y646S
DLBCL_028	BCOR	chrX:39911496 C>T	missense variant	D1712N
DLBCL_028	DDX3X	chrX:41205860 C>G	missense variant	R534G
DLBCL_029	CREBBP	chr16:3788617 C>T	missense variant	R1446H
DLBCL_030	EZH2	chr7:148508728 A>T	missense variant	Y646N

DLBCL_031	EZH2	chr7:148508728 A>G	missense variant	Y646H
DLBCL_031	DDX3X	chrX:41205589 C>T	missense variant	R475C
DLBCL_033	CREBBP	chr16:3843495 G>A	stop gained	R370*
DLBCL_034	DDX3X	chrX:41203603 C>T	missense variant	R326C
DLBCL_039	ETV6	chr12:12038902 C>T	missense variant	R399C
DLBCL_039	MTOR	chr1:11188164 G>A	missense variant	T1977I
DLBCL_039	EZH2	chr7:148508727 T>A	missense variant	Y646F
DLBCL_039	NOTCH1	chr9:139402421 C>T	missense variant	G1166S
DLBCL_039	NOTCH1	chr9:139405696 G>A	missense variant	P832L
DLBCL_039	CDKN2A	chr9:21971051 G>A	missense variant	R103W
DLBCL_040	EZH2	chr7:148508728 A>T	missense variant	Y646N
DLBCL_041	TP53	chr17:7578388 C>T	missense variant	R181H
DLBCL_045	EZH2	chr7:148508728 A>T	missense variant	Y646N
DLBCL_047	MYD88	chr3:38182641 T>C	missense variant	L260P
DLBCL_052	EZH2	chr7:148508728 A>G	missense variant	Y646H
DLBCL_058	CREBBP	chr16:3786766 T>C	missense variant	Y1482C
DLBCL_058	EZH2	chr7:148508727 T>A	missense variant	Y646F
DLBCL_064	CREBBP	chr16:3778440-3778440 TGC>-	inframe deletion	QQ2202-2203Q
DLBCL_065	NOTCH1	chr9:139405696 G>A	missense variant	P832L
DLBCL_066	CREBBP	chr16:3788618 G>A	missense variant	R1446C
DLBCL_066	CREBBP	chr16:3808914 G>A	stop gained	Q1104*
DLBCL_066	MTOR	chr1:11307715 G>A	missense variant	R398C
DLBCL_066	EP300	chr22:41551022 C>T	stop gained	Q1056*
DLBCL_066	EZH2	chr7:148508727 T>G	missense variant	Y646S
DLBCL_066	CDKN2A	chr9:21974765 G>A	missense variant	A21V
DLBCL_070	MYD88	chr3:38182641 T>C	missense variant	L260P
DLBCL_073	DDX3X	chrX:41206206 G>A	stop gained	W570*
DLBCL_074	EZH2	chr7:148508727 T>A	missense variant	Y646F
DLBCL_074	NOTCH1	chr9:139405111 G>A	missense variant	R912W
DLBCL_078	TP53	chr17:7578404 A>G	missense variant	C176R
DLBCL_080	CREBBP	chr16:3828700 C>A	splice donor variant	--
DLBCL_080	EZH2	chr7:148508728 A>G	missense variant	Y646H
DLBCL_081	MYD88	chr3:38182641 T>C	missense variant	L260P
DLBCL_081	NOTCH1	chr9:139399491 C>A	missense variant	S1551I
DLBCL_082	MYD88	chr3:38182641 T>C	missense variant	L260P
DLBCL_082	NOTCH1	chr9:139396835 C>T	missense variant	R1758H
DLBCL_087	CREBBP	chr16:3781324-3781324 AGG>-	inframe deletion	SL1680-1681L
DLBCL_088	EZH2	chr7:148508728 A>T	missense variant	Y646N
DLBCL_090	TP53	chr17:7577536 T>C	missense variant	R249G
DLBCL_090	EZH2	chr7:148508728 A>G	missense variant	Y646H
DLBCL_092	ARID1A	chr1:27105553 C>T	stop gained	R1722*
DLBCL_093	TP53	chr17:7577539 G>A	missense variant	R248W
DLBCL_095	KMT2D	chr12:49425545 G>A	stop gained	Q4315*
DLBCL_095	KMT2D	chr12:49433388 G>A	stop gained	R2687*
DLBCL_095	EP300	chr22:41565529 G>A	missense variant	D1399N
DLBCL_097	EZH2	chr7:148508728 A>T	missense variant	Y646N
DLBCL_102	MYD88	chr3:38182641 T>C	missense variant	L260P

DLBCL_105	TP53	chr17:7577574 T>C	missense variant	Y236C
DLBCL_106	TP53	chr17:7578263 G>A	stop gained	R196*
DLBCL_109	KMT2D	chr12:49426676 G>A	stop gained	Q3938*
DLBCL_109	NOTCH2	chr1:120461101 G>A	missense variant	R1953C
DLBCL_110	KMT2D	chr12:49433060 G>A	stop gained	R2771*
DLBCL_110	KMT2D	chr12:49436599 G>A	stop gained	R1903*
DLBCL_110	CREBBP	chr16:3788618 G>A	missense variant	R1446C
DLBCL_110	EP300	chr22:41572465 G>A	missense variant	R1665H
DLBCL_110	MEF2C	chr5:88100615 T>C	missense variant	T20A
DLBCL_110	EZH2	chr7:148508728 A>T	missense variant	Y646N
DLBCL_114	KMT2D	chr12:49432738 G>A	stop gained	R2801*
DLBCL_114	KMT2D	chr12:49433650 G>A	stop gained	R2635*
DLBCL_114	CREBBP	chr16:3823913 G>A	stop gained	R768*
DLBCL_114	MTOR	chr1:11187857 C>T	missense variant	E2014K
DLBCL_114	NOTCH1	chr9:139391986 C>T	missense variant	A2069T
DLBCL_114	NOTCH1	chr9:139408978 G>A	missense variant	R731W
DLBCL_115	TP53	chr17:7578236 A>G	missense variant	Y205H
DLBCL_117	IRF8	chr16:85953807 C>T	missense variant	R361C
DLBCL_117	TP53	chr17:7577093 C>T	missense variant	R282Q
DLBCL_117	TP53	chr17:7578236 A>G	missense variant	Y205H
DLBCL_117	MTOR	chr1:11193166 C>T	missense variant	A1779T
DLBCL_117	MTOR	chr1:11303294 C>T	missense variant	R430H
DLBCL_117	NOTCH1	chr9:139391907 C>T	missense variant	R2095H
DLBCL_117	NOTCH1	chr9:139399155 C>T	missense variant	R1663Q
DLBCL_117	NOTCH1	chr9:139403510 C>T	missense variant	G995S
DLBCL_117	NOTCH1	chr9:139412302 C>T	missense variant	R448Q
DLBCL_117	CDKN2A	chr9:21971159 C>T	missense variant	G67S
DLBCL_117	CDKN2A	chr9:21974745 C>T	missense variant	V28M
DLBCL_119	NOTCH1	chr9:139404285 C>T	missense variant	G957R
DLBCL_120	TP53	chr17:7577538 C>T	missense variant	R248Q
DLBCL_136	TNFAIP3	chr6:138197178 T>A	stop gained	L227*
DLBCL_137	TP53	chr17:7577142 C>T	missense variant	G266R
DLBCL_138	TP53	chr17:7577127 C>T	missense variant	E271K
DLBCL_140	NOTCH2	chr1:120469121 C>T	splice donor variant	--
DLBCL_142	TP53	chr17:7579310 A>T	splice donor variant	--
DLBCL_143	KMT2D	chr12:49442899 C>A	stop gained	E1337*
DLBCL_143	CREBBP	chr16:3786691 A>G	missense variant	L1507P
DLBCL_143	TP53	chr17:7577079 C>T	missense variant	E287K
DLBCL_143	TP53	chr17:7577142 C>G	missense variant	G266R
DLBCL_143	EP300	chr22:41536164 C>T	missense variant	T594M
DLBCL_143	EZH2	chr7:148508727 T>A	missense variant	Y646F
DLBCL_143	NOTCH1	chr9:139396826 C>T	missense variant	R1761Q
DLBCL_143	BCOR	chrX:39911649 G>A	stop gained	R1661*
DLBCL_143	DDX3X	chrX:41204667 G>A	missense variant	R394H
DLBCL_151	MYD88	chr3:38182641 T>C	missense variant	L260P
DLBCL_160	TP53	chr17:7578262 C>G	missense variant	R196P
DLBCL_167	SPEN	chr1:16256528 C>T	stop gained	R1265*

DLBCL_174	TP53	chr17:7578493 C>T	stop gained	W146*
DLBCL_174	MYD88	chr3:38182641 T>C	missense variant	L260P
DLBCL_177	ETV6	chr12:12037475 G>A	missense variant	R369Q
DLBCL_177	TBL1XR1	chr3:176744183 C>T	missense variant	G499E
DLBCL_177	TBL1XR1	chr3:176750884 G>A	stop gained	R431*
DLBCL_177	MEF2C	chr5:88027605 G>A	missense variant	R269C
DLBCL_177	DDX3X	chrX:41203301 C>T	missense variant	R262C
DLBCL_178	TP53	chr17:7578393 A>T	missense variant	H179Q
DLBCL_179	KMT2D	chr12:49434325 G>A	stop gained	R2410*
DLBCL_179	TP53	chr17:7577114 C>T	missense variant	C275Y
DLBCL_179	TP53	chr17:7578416 C>T	missense variant	V172I
DLBCL_179	MTOR	chr1:11204807 C>T	missense variant	M1590I
DLBCL_179	NOTCH1	chr9:139396823 C>T	missense variant	R1762Q
DLBCL_179	NOTCH1	chr9:139405201 C>T	missense variant	A882T
DLBCL_179	NOTCH1	chr9:139409741 C>T	splice donor variant	--
DLBCL_179	DDX3X	chrX:41205798 T>C	missense variant	V513A
DLBCL_181	CREBBP	chr16:3843447 G>A	stop gained	R386*
DLBCL_186	CREBBP	chr16:3828700 C>T	splice donor variant	--
DLBCL_186	TP53	chr17:7577538 C>T	missense variant	R248Q
DLBCL_186	TBL1XR1	chr3:176755899 T>C	missense variant	D370G
DLBCL_186	MYD88	chr3:38182641 T>C	missense variant	L260P
DLBCL_186	NOTCH1	chr9:139399264 G>A	missense variant	R1627C
DLBCL_186	NOTCH1	chr9:139413193 C>T	missense variant	G317S
DLBCL_187	EZH2	chr7:148508727 T>G	missense variant	Y646S
DLBCL_189	TP53	chr17:7577539 G>C	missense variant	R248G
DLBCL_194	NOTCH1	chr9:139405696 G>A	missense variant	P832L
DLBCL_195	EZH2	chr7:148508727 T>A	missense variant	Y646F
DLBCL_197	MYD88	chr3:38182641 T>C	missense variant	L260P
DLBCL_202	EZH2	chr7:148508727 T>A	missense variant	Y646F
DLBCL_205	EZH2	chr7:148508728 A>T	missense variant	Y646N
DLBCL_212	TP53	chr17:7577094 G>A	missense variant	R282W
DLBCL_212	CDKN2A	chr9:21971186 G>A	stop gained	R58*
DLBCL_221	CREBBP	chr16:3778440-3778440 TGC>-	inframe deletion	QQ2202-2203Q
DLBCL_227	NOTCH1	chr9:139396835 C>T	missense variant	R1758H
DLBCL_235	ETV6	chr12:12038902 C>T	missense variant	R399C
DLBCL_235	CREBBP	chr16:3789685 G>A	stop gained	R1392*
DLBCL_235	MTOR	chr1:11188524 C>T	missense variant	R1966Q
DLBCL_235	EP300	chr22:41564765 C>T	stop gained	R1356*
DLBCL_235	TBL1XR1	chr3:176751990 C>T	missense variant	A416T
DLBCL_236	TP53	chr17:7577505 T>C	missense variant	D259G
DLBCL_239	TP53	chr17:7578406 C>T	missense variant	R175H
DLBCL_240	EZH2	chr7:148508728 A>T	missense variant	Y646N
DLBCL_241	TP53	chr17:7578263 G>A	stop gained	R196*
DLBCL_242	KMT2D	chr12:49420578 C>T	stop gained	W5057*
DLBCL_242	CREBBP	chr16:3778440-3778440 TGC>-	inframe deletion	QQ2202-2203Q
DLBCL_242	TP53	chr17:7578470 C>T	missense variant	G154S
DLBCL_242	TP53	chr17:7578500 G>A	stop gained	Q144*

DLBCL_242	CDKN2A	chr9:21970951 C>T	missense variant	G136D
DLBCL_242	BCOR	chrX:39913212 C>T	missense variant	D1635N
DLBCL_242	DDX3X	chrX:41203501 C>T	stop gained	R292*
DLBCL_249	EZH2	chr7:148508727 T>A	missense variant	Y646F
DLBCL_261	EZH2	chr7:148508728 A>G	missense variant	Y646H
DLBCL_265	DDX3X	chrX:41205589 C>T	missense variant	R475C
DLBCL_266	DDX3X	chrX:41205590 G>A	missense variant	R475H
DLBCL_268	CDKN2A	chr9:21974681 A>G	missense variant	I49T
DLBCL_269	EZH2	chr7:148508727 T>A	missense variant	Y646F
DLBCL_272	TP53	chr17:7577539 G>A	missense variant	R248W
DLBCL_272	MTOR	chr1:11264706 G>A	missense variant	R1286W
DLBCL_272	EZH2	chr7:148508727 T>G	missense variant	Y646S
DLBCL_272	DDX3X	chrX:41205855 C>T	missense variant	T532M
DLBCL_274	KMT2D	chr12:49416372 C>T	splice donor variant	--
DLBCL_274	KMT2D	chr12:49426772-49426772 GCT>-	inframe deletion	QL3905-3906L
DLBCL_274	CREBBP	chr16:3779038 G>A	stop gained	R2004*
DLBCL_274	CREBBP	chr16:3789685 G>A	stop gained	R1392*
DLBCL_274	TP53	chr17:7577117 A>G	missense variant	V274A
DLBCL_274	TP53	chr17:7578388 C>T	missense variant	R181H
DLBCL_274	TP53	chr17:7579592 T>C	splice acceptor variant	--
DLBCL_274	MTOR	chr1:11187790 C>T	missense variant	R2036H
DLBCL_274	MTOR	chr1:11190804 C>T	missense variant	E1799K
DLBCL_274	NOTCH2	chr1:120458147 G>A	stop gained	R2400*
DLBCL_274	NOTCH2	chr1:120461100 C>T	missense variant	R1953H
DLBCL_274	NOTCH2	chr1:120462900 G>A	stop gained	Q1811*
DLBCL_274	BCL10	chr1:85733524 G>A	missense variant	T163M
DLBCL_274	EP300	chr22:41513352 C>T	stop gained	R86*
DLBCL_274	TBL1XR1	chr3:176755899 T>C	missense variant	D370G
DLBCL_274	EZH2	chr7:148508728 A>G	missense variant	Y646H
DLBCL_274	NOTCH1	chr9:139393636 G>A	missense variant	R2004C
DLBCL_274	NOTCH1	chr9:139399480 C>T	missense variant	E1555K
DLBCL_274	NOTCH1	chr9:139409753 G>A	missense variant	P668L
DLBCL_274	NOTCH1	chr9:139412607 C>T	missense variant	V413M
DLBCL_274	BCOR	chrX:39911649 G>A	stop gained	R1661*
DLBCL_274	DDX3X	chrX:41193545 C>T	stop gained	Q14*
DLBCL_274	DDX3X	chrX:41205628 C>T	missense variant	R488C
DLBCL_275	KMT2D	chr12:49433060 G>A	stop gained	R2771*
DLBCL_275	KMT2D	chr12:49436599 G>A	stop gained	R1903*
DLBCL_275	CREBBP	chr16:3788618 G>A	missense variant	R1446C
DLBCL_275	EZH2	chr7:148508728 A>T	missense variant	Y646N
DLBCL_280	CREBBP	chr16:3779862 C>G	missense variant	C1729S
DLBCL_280	CREBBP	chr16:3900498 G>A	stop gained	Q200*
DLBCL_280	TP53	chr17:7577022 G>A	stop gained	R306*
DLBCL_280	NOTCH2	chr1:120461100 C>T	missense variant	R1953H
DLBCL_280	NOTCH2	chr1:120464895 C>T	missense variant	R1726H
DLBCL_280	NOTCH2	chr1:120506251 G>A	missense variant	R621C
DLBCL_280	TBL1XR1	chr3:176750884 G>A	stop gained	R431*

DLBCL_280	MEF2C	chr5:88027589 C>T	missense variant	R274Q
DLBCL_280	BCOR	chrX:39923601 G>A	stop gained	R1164*
DLBCL_280	DDX3X	chrX:41205629 G>A	missense variant	R488H
DLBCL_280	DDX3X	chrX:41205852 G>A	missense variant	R531H
DLBCL_286	TP53	chr17:7577538 C>T	missense variant	R248Q
DLBCL_288	BCOR	chrX:39911649 G>A	stop gained	R1661*
DLBCL_289	EZH2	chr7:148508727 T>A	missense variant	Y646F
DLBCL_290	IRF8	chr16:85942659 A>G	missense variant	T80A
DLBCL_290	TP53	chr17:7577572 T>C	missense variant	M237V
DLBCL_301	CREBBP	chr16:3781324-3781324 AGG>-	inframe deletion	SL1680-1681L
DLBCL_301	CREBBP	chr16:3788617 C>T	missense variant	R1446H
DLBCL_304	EZH2	chr7:148508728 A>T	missense variant	Y646N
DLBCL_307	EZH2	chr7:148508727 T>A	missense variant	Y646F
DLBCL_307	BCOR	chrX:39914683 G>A	missense variant	T1560M
DLBCL_307	DDX3X	chrX:41205842 C>T	missense variant	R528C
DLBCL_309	KMT2D	chr12:49444035-49444035 CAGGGCTGGGG>-	frameshift variant	APAL1109-1112X
DLBCL_309	BCL6	chr3:187451339 G>A	missense variant	T48M
DLBCL_310	CREBBP	chr16:3779704 C>T	missense variant	A1782T
DLBCL_310	CREBBP	chr16:3795277 C>T	splice donor variant	--
DLBCL_310	TP53	chr17:7577586 A>G	missense variant	I232T
DLBCL_310	TP53	chr17:7578401 G>A	missense variant	P177S
DLBCL_310	MEF2C	chr5:88027605 G>A	missense variant	R269C
DLBCL_310	TRRAP	chr7:98606065 C>T	missense variant	L3607F
DLBCL_310	NOTCH1	chr9:139405180 C>T	missense variant	G889S
DLBCL_310	BCOR	chrX:39913212 C>T	missense variant	D1635N
DLBCL_316	NOTCH1	chr9:139405696 G>A	missense variant	P832L
DLBCL_326	CDKN2A	chr9:21974721 C>A	missense variant	A36S
DLBCL_327	KMT2D	chr12:49427489 G>A	stop gained	Q3667*
DLBCL_327	KMT2D	chr12:49433394 G>A	stop gained	R2685*
DLBCL_327	MTOR	chr1:11168311 C>T	missense variant	V2521I
DLBCL_327	MTOR	chr1:11188578 G>A	missense variant	T1948M
DLBCL_327	MTOR	chr1:11210213 G>A	missense variant	R1514W
DLBCL_327	BCOR	chrX:39911649 G>A	stop gained	R1661*
DLBCL_327	DDX3X	chrX:41203531 G>A	missense variant	G302S
DLBCL_328	CREBBP	chr16:3786703 T>C	missense variant	Y1503C
DLBCL_329	KMT2D	chr12:49420460 G>A	stop gained	R5097*
DLBCL_329	TP53	chr17:7577511 A>T	missense variant	L257Q
DLBCL_331	MYD88	chr3:38182641 T>C	missense variant	L260P
DLBCL_331	CDKN2A	chr9:21971186 G>A	stop gained	R58*

Suppl Table S3.												
**Genes**	***Sex***			***B symptoms***			***Bulky mass***			***Clinical stage****		
	Female (%)	***Male (%)***	***Fisher test (p-value)***	***B symptoms (%)***	***No B symptoms (%)***	***Fisher test (p-value)***	***Bulky mass (%)***	***No bulky mass (%)***	***Fisher test (p-value)***	***Early stage (I-II) (%)***	***Advanced stage (III-IV) (%)***	***Fisher test (p-value)***
ARID1A	2.15	0.00	0.251	0.00	1.50	1.000	0.00	1.96	0.303	0.75	1.96	0.402
BCL10	1.08	0.00	0.503	0.00	0.75	1.000	1.20	0.00	0.449	0.75	0.00	0.724
BCL6	1.08	1.09	0.503	0.00	1.50	1.000	2.41	0.00	0.200	1.49	0.00	0.524
BCOR	8.60	2.17	**0.042**	3.85	6.02	0.257	6.02	4.90	0.239	5.22	5.88	0.271
CDKN2A	3.23	6.52	0.161	1.92	6.02	0.177	4.82	4.90	0.267	5.97	1.96	0.185
CIITA	1.08	0.00	0.503	0.00	0.75	1.000	0.00	0.98	0.551	0.00	1.96	0.276
CREBBP	16.13	10.87	0.100	19.23	11.28	0.068	16.87	10.78	0.084	13.43	13.73	0.188
DDX3X	9.68	6.52	0.158	7.69	8.27	0.235	8.43	7.84	0.210	10.45	1.96	**0.040**
EP300	3.23	4.35	0.276	5.77	3.01	0.210	6.02	1.96	0.114	4.48	1.96	0.279
ETV6	1.08	3.26	0.247	0.00	3.01	1.000	0.00	3.92	0.090	2.24	1.96	0.423
EZH2	23.66	13.04	**0.027**	26.92	15.04	**0.030**	25.30	12.75	**0.014**	22.39	7.84	**0.011**
IRF8	1.08	1.09	0.503	1.92	0.75	0.406	0.00	1.96	0.303	1.49	0.00	0.524
KMT2D	12.90	4.35	**0.025**	11.54	7.52	0.149	13.25	4.90	**0.029**	8.96	7.84	0.228
MEF2C	1.08	4.35	0.152	3.85	2.26	0.297	3.61	1.96	0.277	2.99	1.96	0.383
MTOR	4.30	6.52	0.206	7.69	4.51	0.184	4.82	5.88	0.245	5.97	3.92	0.263
MYD88	3.23	10.87	**0.030**	3.85	8.27	0.161	3.61	9.80	0.063	6.72	7.84	0.235
NOTCH1	9.68	10.87	0.184	13.46	9.02	0.136	9.64	10.78	0.187	10.45	9.80	0.213
NOTCH2	4.30	4.35	0.279	1.92	5.26	0.222	7.23	1.96	0.067	4.48	3.92	0.314
SPEN	0.00	1.09	0.497	0.00	0.75	1.000	0.00	0.98	0.551	0.00	1.96	0.276
TBL1XR1	3.23	3.26	0.318	0.00	4.51	1.000	2.41	3.92	0.282	2.99	3.92	0.319
TNFAIP3	0.00	1.09	0.497	0.00	0.75	1.000	0.00	0.98	0.551	0.00	1.96	0.276
TP53	19.35	18.48	0.147	19.23	18.80	0.164	19.28	18.63	0.148	23.13	7.84	**0.009**
TRRAP	0.00	1.09	0.497	0.00	0.75	1.000	0.00	0.98	0.551	0.00	1.96	0.276

* Clinical stage is according to the Ann Arbor staging system. *p*-value < 0.05 are represented in bold

Supplementary Table S4.

**Genes**	**Extranodal disease**			**IPI score**			**Cell of Origin**			**Chemotherapy response**		
	**2 extranodal sites (%)**	**1 extranodal sites (%)**	**Fisher test (p-value)**	**High IPI score (%)**	**Low IPI score (%)**	**Fisher test (p-value)**	**GCB (%)**	**Non GCB (%)**	**Fisher test (p-value)**	**Complete chemotherapy response (%)**	**Uncomplete chemotherapy response (%)**	**Fisher test (p-value)**
ARID1A	0.00	1.59	0.463	1.00	1.18	0.499	1.59	0.00	0.619	0.81	2.27	0.389
BCL10	0.00	0.79	0.681	0.00	1.18	0.459	0.79	0.00	0.788	0.81	0.00	1.000
BCL6	0.00	1.59	0.463	2.00	0.00	0.291	1.59	0.00	0.619	0.81	0.00	1.000
BCOR	5.08	5.56	0.272	4.00	7.06	0.170	5.56	2.94	0.322	5.65	6.82	0.265
CDKN2A	8.47	3.17	0.087	7.00	2.35	0.100	3.17	8.82	0.128	4.84	4.55	0.318
CIITA	0.00	0.79	0.681	0.00	1.18	0.459	0.79	0.00	0.788	0.81	0.00	1.000
CREBBP	8.47	15.87	0.076	13.00	14.12	0.166	15.87	2.94	**0.030**	12.90	11.36	0.206
DDX3X	8.47	7.94	0.222	8.00	8.24	0.211	7.94	2.94	0.211	8.87	9.09	0.238
EP300	3.39	3.97	0.319	4.00	3.53	0.295	3.97	2.94	0.392	4.84	2.27	0.298
ETV6	5.08	0.79	0.087	2.00	2.35	0.374	0.79	5.88	0.106	2.42	0.00	1.000
EZH2	6.78	23.81	**0.003**	21.00	15.29	0.093	23.81	2.94	**0.003**	19.35	18.18	0.176
IRF8	1.69	0.79	0.437	1.00	1.18	0.499	0.79	2.94	0.337	1.61	0.00	1.000
KMT2D	5.08	10.32	0.120	9.00	8.24	0.203	10.32	5.88	0.214	8.87	6.82	0.239
MEF2C	3.39	2.38	0.326	2.00	3.53	0.286	2.38	5.88	0.223	4.03	0.00	1.000
MTOR	5.08	5.56	0.272	3.00	8.24	0.079	5.56	2.94	0.322	7.26	0.00	1.000
MYD88	13.56	3.97	**0.017**	6.00	8.24	0.190	3.97	14.71	**0.030**	7.26	4.55	0.249
NOTCH1	10.17	10.32	0.204	10.00	10.59	0.189	10.32	8.82	0.252	10.48	2.27	0.064
NOTCH2	3.39	4.76	0.289	2.00	7.06	0.074	4.76	5.88	0.310	4.84	2.27	0.298
SPEN	0.00	0.79	0.681	1.00	0.00	0.541	0.79	0.00	0.788	0.81	0.00	1.000
TBL1XR1	5.08	2.38	0.206	1.00	5.88	0.064	2.38	2.94	0.421	4.84	0.00	1.000
TNFAIP3	1.69	0.00	0.319	0.00	1.18	0.459	0.00	2.94	0.213	0.81	0.00	1.000
TP53	18.64	19.05	0.159	20.00	17.65	0.138	19.05	20.59	0.186	18.55	22.73	0.142
TRRAP	1.69	0.00	0.319	0.00	1.18	0.459	0.00	2.94	0.213	0.81	0.00	1.000

IPI: International prognostic index; GCB: Germinal center B cell. *p*-values z 0.05 are represented in bold.

Supplementary table S5. Univariate analysis for clinical features and genes with driver mutations

	Univariate analysis for OS				Univariate analysis for RFS			
	**β**	**HR (95% CI for HR)**	**Wald test**	***p*-value**	**β**	**HR (95% CI for HR)**	**Wald test**	***p*-value**
**Age**	0.04	1.00 (1.00-1.10)	12.00	**0.001**	0.004	1.00 (0.98-1.00)	0.16	0.690
**Sex**	-0.02	0.99 (0.56-1.70)	0.00	0.960	-0.17	0.84 (0.47-1.50)	0.32	0.570
**ECOG**	0.55	1.70 (1.20-2.50)	8.30	**0.004**	0.57	1.80 (1.20-2.60)	8.60	**0.003**
**B symptoms**	0.34	1.40(0.78-2.50)	1.30	0.250	0.45	1.60 (0.85-2.90)	2.10	0.150
**Bulky mass**	0.38	1.50 (0.84-2.60)	1.80	0.180	0.97	2.60 (1.40-4.90)	9.60	**0.002**
**Extranodal disease**	-0.44	0.64 (0.33-1.20)	1.80	0.180	-0.33	0.72 (0.37-1.40)	0.96	0.330
**Clinical Stage**	0.38	1.50 (1.10-2.00)	5.30	**0.021**	0.35	1.40 (1.00-2.00)	4.50	**0.034**
**IPI score**	0.35	1.40 (1.10-1.80)	7.60	**0.006**	0.36	1.40 (1.10-1.80)	7.30	**0.007**
**Cell of origin**	-0.06	0.94 (0.67-1.30)	0.12	0.730	0.06	1.10 (0.75-1.50)	0.10	0.750
***TP53* mutation**	0.12	1.10 (0.56-2.30)	0.11	0.730	0.41	1.50 (0.76-3.00)	1.40	0.240
***EZH2* mutation**	-0.62	0.54 (0.21-1.40)	1.70	0.190	0.06	1.10 (0.49-2.30)	0.02	0.880
***CREBBP* mutation**	0.30	1.40 (0.63-2.90)	0.61	0.430	0.31	1.40 (0.61-3.00)	0.55	0.460
***NOTCH1* mutation**	-0.22	0.81 (0.29-2.20)	0.17	0.680	-1.60	0.20 (0.03-1.40)	2.50	0.110
***KMT2D* mutation**	-0.36	0.69 (0.22-2.20)	0.37	0.540	0.30	1.40 (0.54-3.40)	0.41	0.520
***DDX3X* mutation**	-0.70	0.50 (0.12-2.00)	0.94	0.330	0.14	1.20 (0.41-3.20)	0.07	0.790
***MYD88* mutation**	0.14	1.10 (0.41-3.20)	0.07	0.790	-0.07	0.94 (0.29-3.00)	0.01	0.910
***BCOR* mutation**	0.19	1.20 (0.38-3.90)	0.11	0.750	0.56	1.80 (0.62-4.90)	1.10	0.290
***MTOR* mutation**	-1.10	0.34 (0.05-2.40)	1.20	0.280	-17.00	3.7e-08 (0.00-Inf)	0.00	1.000
***CDKN2A* mutation**	-0.86	0.42 (0.06-3.10)	0.73	0.390	0.41	1.50 (0.47-4.90)	0.46	0.500
***NOTCH2* mutation**	-0.03	0.97 (0.24-4.00)	0.00	0.970	-0.69	0.50 (0.07-3.60)	0.47	0.490
***EP300* mutation**	-0.71	0.49 (0.07-3.60)	0.49	0.480	-17.00	3.8e-08 (0.00-Inf)	0.00	1.000
***TBL1XR1* mutation**	-17.00	3.9e-08 (0.00-Inf)	0.00	1.000	-17.00	3.9e-08 (0.00-Inf)	0.00	1.000
***MEF2C* mutation**	-17.00	3.9e-08 (0.00-Inf)	0.00	1.000	-17.00	3.9e-08 (0.00-Inf)	0.00	1.000
***ETV6* mutation**	-16.00	1.1e-07 (0.00-Inf)	0.00	1.000	-17.00	4e-08 (0.00-Inf)	0.00	1.000
***ARID1A* mutation**	1.50	4.30 (1.00-18.00)	4.10	**0.043**	0.79	2.20 (0.30-16.00)	0.61	0.430
***BCL6* mutation**	-15.00	3e-07 (0.00-Inf)	0.00	1.000	-15.00	3e-07 (0.00-Inf)	0.00	1.000
***IRF8* mutation**	-16.00	1.1e-07 (0.00-Inf)	0.00	1.000	-16.00	1.1e-07 (0.00-Inf)	0.00	1.000
***BCL10* mutation**	-15.00	3e-07 (0.00-Inf)	0.00	1.000	-15.00	3e-07 (0.00-Inf)	0.00	1.000
***CIITA* mutation**	1.20	3.40 (0.46-24)	1.40	0.230	1.40	4.00 (0.55-29.00)	1.90	0.170
***SPEN* mutation**	-15.00	3e-07 (0.00-Inf)	0.00	1.000	-15.00	3e-07 (0.00-Inf)	0.00	1.000
***TNFAIP3* mutation**	-15.00	3e-07 (0.00-Inf)	0.00	1.000	-15.00	3e-07 (0.00-Inf)	0.00	1.000
***TRRAP* mutation**	-15.00	3e-07 (0.00-Inf)	0.00	1.000	-15.00	3e-07 (0.00-Inf)	0.00	1.000

HR: Hazard ratio; OS: overall survival; RFS: Relapse-free survival
